# Fabrication, Experiments, and Analysis of an LBM Additive-Manufactured Flexure Parallel Mechanism

**DOI:** 10.3390/mi9110572

**Published:** 2018-11-05

**Authors:** Huaxian Wei, Li Wang, Xiaodong Niu, Jian Zhang, Alessandro Simeone

**Affiliations:** 1 Department of Mechatronic Engineering, College of Engineering, Shantou University, Shantou 515063, China; 16lwang4@stu.edu.cn (L.W.); xdniu@stu.edu.cn (X.N.); jianzhang@stu.edu.cn (J.Z.); simeone@stu.edu.cn (A.S.); 2 Intelligent Manufacturing Key Laboratory of Ministry of Education, Shantou University, Shantou 515063, China; 3 Shantou Ray-Bonus Additive Manufacture Research Institute, Shantou 515063, China

**Keywords:** flexure hinges, laser beam melting, additive manufacturing, stiffness, manufacturing error, 316L

## Abstract

Additive manufacturing technology has advantages for realizing complex monolithic structures, providing huge potential for developing advanced flexure mechanisms for precision manipulation. However, the characteristics of flexure hinges fabricated by laser beam melting (LBM) additive manufacturing (AM) are currently little known. In this paper, the fabrication and characterization of a flexure parallel mechanism through the LBM process are reported for the first time to demonstrate the development of this technique. The geometrical accuracy of the additive-manufactured flexure mechanism was evaluated by three-dimensional scanning. The stiffness characteristics of the flexure mechanism were investigated through finite element analysis and experimental tests. The effective hinge thickness was determined based on the parameters study of the flexure parallel mechanism. The presented results highlight the promising outlook of LBM flexure parts for developing novel nanomanipulation platforms, while additional attention is required for material properties and manufacturing errors.

## 1. Introduction

Flexure mechanisms (or compliant mechanisms) have been widely utilized as the basic platforms in ultra-precise manipulation, such as semiconductor manufacturing, microassembly, and atomic force microscopy [1,2,3,4]. Unlike traditional mechanisms, flexure mechanisms comprise monolithic flexure hinges instead of traditional pivots, which eliminate mechanical clearance, friction, and lubrication issues. Consequently, flexure mechanisms have the capabilities of infinite resolution of motion. In addition, flexure mechanisms can realize monolithic and compact designs for high speed and vacuum applications [5].

Currently, most of flexure mechanisms are fabricated through computer numerical controlled (CNC) milling or wire electrical discharge machining (wire-EDM), since the flexure hinges within the structure are generally slim and weak [6]. The manufacturing technique is an important issue especially when developing complex flexure mechanisms. For example, many existing parallel flexure mechanisms are generally divided into subparts that are fabricated separately and eventually assembled together [7,8]. In addition, assembling generally introduces complicated issues including assembling error, stiffness variation, and volume increment [9]. Nowadays, the demand of light-weight and nonassembly products is increasing in many advanced technologies, such as in surgical and aerospace applications, where compact and complex flexure mechanisms are required [10]. The advantages of freeform fabrication of the additive manufacturing (AM or 3D printing) provide great potential for developing complex flexure mechanisms.

In the existing literature, many researchers have developed prototypes of flexure mechanisms through 3D printing using plastic materials due to their widespread use both in academia and industry [11,12]. However, plastic products suffer disadvantages such as low stiffness, short strength life, and creep deformation, which limit their application in engineering [13]. For instance, surgical products are generally more prone to blood contamination and are only suitable for single use [10]. Besides, most 3D-printed plastic products nowadays are not suitable for radiate or high-temperature environments [14]. On the contrary, metallic products are less sensitive to environment and have better reliability. However, metallic additive-manufactured flexure mechanisms are currently rarely studied. Merriam and Howell designed a monolithic additive-manufactured titanium flexure mechanism for space pointing [14,15,16]. Pham developed an additive-manufactured parallel flexure mechanism for high-precision manipulation [17]. Fiaz reported the design, fabrication, and testing of an additive-manufactured, flexure-based XY nanopositioning stage [18]. The available literature of AM flexures only focuses on electron beam melting (EBM) additive manufacturing technology and Ti-6Al-4V material. In addition, it has been observed that the EBM additive-manufactured slender parts have low effective thickness due to the high surface roughness.

In the past decade, extensive research has been proposed on investigating the nature of the AM processes contributing to the transformation from rapid prototyping to freeform manufacturing applications [19,20,21]. Among the most popular processes for the AM of metals, laser beam melting (LBM) and EBM have greatly advanced with respect to both the process and feedstock in recent years [22]. Both processes have advantages and disadvantages due to the distinguishing thermal conditions when melting the material [23]. So far, only a few studies have reported AM metal flexure parts and are limited to EBM and Ti-6Al-4V materials, and there is a gap in the literature regarding flexure mechanisms fabricated by LBM technology. Hence, the main focus of this paper is introducing LBM in flexure mechanisms. The LBM process has been selected as a potential process for next-generation flexure mechanisms with two considerations:

(a). The LBM process can produce better surface quality than the EBM process, especially for structures with small features [24,25].
(b). The LBM process has a large range of building materials [26]. 316L has been widely investigated and utilized for research and engineering. The cost of fabricating 316L parts through LBM is competitive compared with that of traditional manufacturing, especially for small batch sizes.

Whilst most existing applications of AM metallic parts serve as structural members or nonstructural assemblies, the AM flexure parts provide more functional objectives as mechanisms. Therefore, the performances of an LBM flexure sample are investigated from an application-oriented perspective in this article. Flexure mechanisms achieve precise motions through the deformation of the flexure hinges that are contained within the structure when subject to external forces. Consequently, the stiffness characteristics that relate the actuating forces to the corresponding displacements represent an important issue when designing flexure mechanisms. The material properties, such as Young’s modulus, are clear for products made by traditional manufacturing technologies, such as molding and extruding, but not for the additive-manufactured flexure parts in particular [27,28]. The manufacturing errors of the additive-manufactured parts are more critical due to the layer-by-layer deposition process. All these factors should be considered for the development of flexure mechanisms for precision applications and hence have been focused on for the first time with respect to the LBM process.

The main contributions of this article are the fabrication, experiments, and analysis of an LBM additive-manufactured flexure parallel mechanism, which could serve as first-step verification for the development of complex flexure mechanisms. The design, fabrication, and experimental methods of the additive-manufactured flexure mechanism are described in the next section. Then, the experimental results are analyzed comprehensively in Section 3. Finally, discussions and conclusions are given in Section 4 and Section 5, respectively.

## 2. Materials and Methods

### 2.1. Flexure Parallel Mechanism

Flexure mechanisms are composed of rigid blocks and flexure hinges. The flexure hinges differentiate the characteristics of the flexure mechanisms. Among the many kinds of flexure hinges, the widespread leaf-type flexure hinges were considered in this study due to the relatively critical geometry for the LBM process. Leaf-type flexure hinges have the same structure as cantilevers with a rectangular cross section. When the length is much bigger than the width and thickness, leaf-type flexure hinges are relatively flexible along the bending direction but stiff along the other directions.

**Figure 1:**
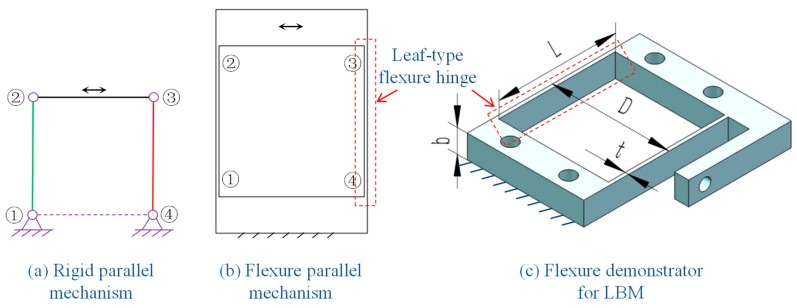
(**a**) Illustration of the equivalent rigid parallel mechanism for ((**b**) top view) the flexure parallel mechanism and (**c**) the 3D model of the flexure demonstrator for laser beam melting (LBM) with the key dimensional parameters denoted as: the length of the flexure hinges *L*, the thickness of the flexure hinges *t*, the width of the flexure hinges *b*, and the distance between the flexure hinges *D.*

With the aim of evaluating the performance of a new application in a manufacturing system, the choice of using a simple geometry was considered. Rather than fabricating a single flexure hinge, the flexure parallel mechanism was selected as the flexure demonstrator for LBM to simplify the motivation and measurement of motion. Such a mechanism is derived from a rigid parallel mechanism having a dominating single degree of freedom, as shown in Figure 1. In addition, the relatively complex structure of the flexure parallel mechanism which comprises two leaf flexure hinges and two rigid blocks further help to investigate geometrical characteristics of the LBM process for flexure mechanisms. Moreover, the leaf-type flexure hinges and the parallel flexure mechanisms have been utilized in previous studies for the EBM process [14,16,17,18], which help to provide a comparison for the research detailed in this paper. 

Compared to the rigid parallel mechanisms, one of the main advantages of flexure parallel mechanisms is the capability of motion resolution up to the nanometer scale due to the elimination of mechanical clearance by utilizing flexure hinges instead of rigid pivots. In operating conditions, the flexure parallel mechanism can serve as a pure translational guide when one of the rigid blocks is fixed and the other rigid block is actuated externally. The actuating force *F* and the corresponding displacement *x* can be related by the stiffness, as according to Equation (1):

(1) $$\;K=\frac{F}{x}\;$$

### 2.2. Fabrication, Measurement, and Experiments

The LBM process was accomplished utilizing a commercial AM machine (FS271M, Farsoon Technologies, Changsha, China). The machine has a building volume of 275 × 275 × 320 mm^3^, adjustable laser spot between 70 and 200 μm, and a 500-W Yb-fiber laser source. 316L stainless-steel powder was selected as the fabrication material considering its widespread utilization and economic accessibility. The powder size ranges from 20 to 60 μm with an average size of 35 μm. The powder composition of the 316L material is given in Table 1.

**Figure 2:**
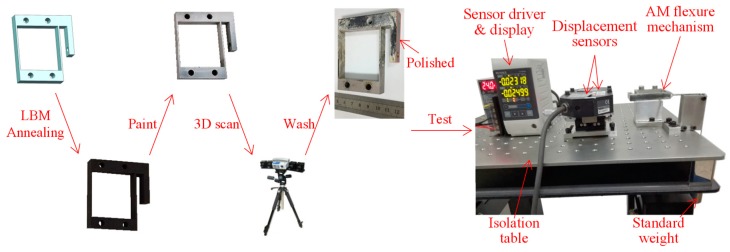
Procedure for processing the LBM additive-manufactured flexure parallel mechanism, with the photo of the sample in each step and the experimental apparatus for stiffness and motion tests.

A flexure parallel mechanism was fabricated through the LBM process. Figure 2 illustrates the overall procedure for processing the presented LBM flexure sample during the research. The presented flexure sample was LBM fabricated and annealed firstly before cutting off from the building plate. Then, the sample was carefully painted with spray paint for 3D scanning. The coat was evenly around several micrometers and removed after 3D scanning by washing with water and therefore had little influence on the geometry and characteristics of the sample. The side face of the rigid block in the output direction was polished for displacement measurement.

The main geometrical dimensions of the demonstrator were: *t* = 1.5 mm, *b* = 10 mm, *L* = 50 mm, and *D* = 50 mm. The building direction was along the width of the mechanism, i.e., *b* as shown in Figure 1. In such a building direction, the fabrication can achieve best quality because no support is required. The LBM process was carried out under nitrogen protection with the main process parameters including: a layer thickness of 0.03 mm, a fill laser power of 225 W, a fill speed of 1000 mm/s, and a fill distance of 0.09 mm. The as-built part was subjected to an annealing process before being cut off from the building plate to eliminate the residual stress. The annealing process was carried out with nitrogen protection, under which the samples were treated at 899 °C for 2 h followed by furnace cooling.

These processing parameters were derived and optimized from various kinds of experiments and tests that have proved to be effective for most structural parts. These experiments and tests generally follow the framework of “structure-property-processing-performance”, which mainly includes single-track melting experiments, artifact tests, mechanical properties tests, etc. The LBM 316L parts fabricated from this machine under the same processing parameters achieved density over 99%, yield strength of 550 ± 50 MPa, tensile strength over 600 MPa, and elongation after fracture around 35%.

To evaluate the geometry of the LBM additive-manufactured flexure parallel mechanism, the dimension of the demonstrator was measured through a three-dimensional scanner (3DX-IV, Dimenxun, Huizhou, China) which had an accuracy of ±0.015 mm. The measured results were compared to the 3D model of the mechanism for the evaluation of geometrical characteristics.

As shown in Figure 2, a test bench was set up to assess the stiffness characteristics and motion accuracy of the LBM additive-manufactured flexure parallel mechanism. During the experiment, one rigid block of the flexure parallel mechanism was fixed to the bench, while the other block was connected to standard weights through a wire which was perpendicular to the flexure hinges. By varying the standard weight from 0 to 800 g and then back to 0 g with a step of 200 g, the displacements of the free block were measured by two laser displacement sensors (LK-G30, Keyence, Osaka, Japan) with a distance of 29.25 mm between the two measuring laser spots. The measurement was repeated for five times to obtain the average results.

## 3. Results

### 3.1. Analysis of Geometrical Measurements

**Figure 3:**
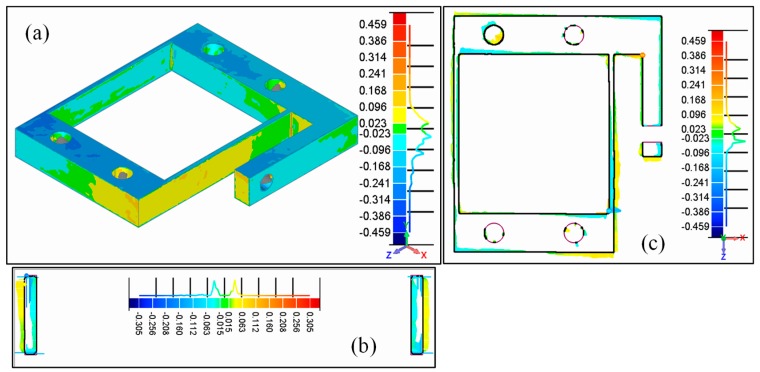
Geometrical analysis of the LBM additive-manufactured flexure parallel mechanism through 3D scanning: (**a**) 3D deviation; (**b**) cross section at the middle of hinge length *L*; (**c**) cross section at the middle of the hinge width *b* (mm).

Figure 3 shows the deviation of geometry between the as-built flexure parallel mechanism measured by 3D scanning and the designed 3D model to evaluate the overall fabrication error of the LBM process for the flexure mechanism. It can be observed that three-dimensional manufacturing errors exist both on the flexure hinges and the rigid blocks of the LBM additive-manufactured flexure parallel mechanism. Statistical analysis was used to characterize the manufacturing error of the obtained LBM flexure parts. It was found that 77% of the geometry deviations were within ±0.093 mm and 93% of the geometry deviations were within ±0.165 mm. By evaluating the measured points on the flexure hinges solely, it was further found that the manufacturing error on the flexure hinges was better, most of which was less than ±0.063 mm. In addition, the deviation along the width *b* (or the building direction) was larger than that along the thickness *t*. This was mainly caused by the wire-EDM process when cutting the parts off the building plate.

Applying thickness evaluation and 2D dimension detection, the measured geometrical dimensions of the LBM additive-manufactured flexure parallel mechanism were determined to be: *t* = 1.505 mm, *b* = 9.75 mm, *L* = 50.069 mm, and *D* = 50 mm. This means that the 3D-detected hinge thickness and hinge distance results increased while the hinge width results decreased with reference to the designed values. However, it should be noted that LBM additive-manufactured flexure hinges introduce manufacturing errors not only on the geometrical parameters but also on the position and orientation of the flexure hinges and the rigid blocks. This is mainly caused by the layer-by-layer process of the additive manufacturing and the thermal stress caused by the high cooling rate during the LBM process.

### 3.2. Analysis of Stiffness Experiments

**Figure 4:**
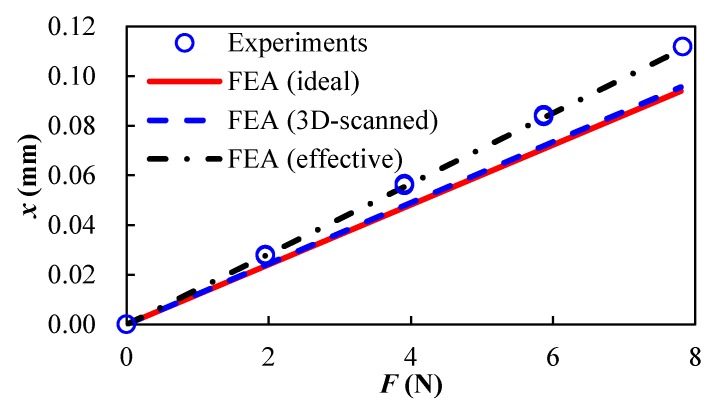
Force-displacement results of the LBM additive-manufactured flexure parallel mechanism by experimental tests and finite element analysis.

As shown in Figure 4, the displacements of the moving block of the LBM additive-manufactured flexure parallel mechanism were obtained during the stiffness experiments. The motivating forces were calculated from the weight of the standard weight using a gravity factor of 9.78 N/Kg. The experimental stiffness was calculated utilizing Equation (1) for each step when varying the standard weight, and the average stiffness of the seven steps was *K*_experiment_ = 68.49 N/mm.

**Figure 5:**
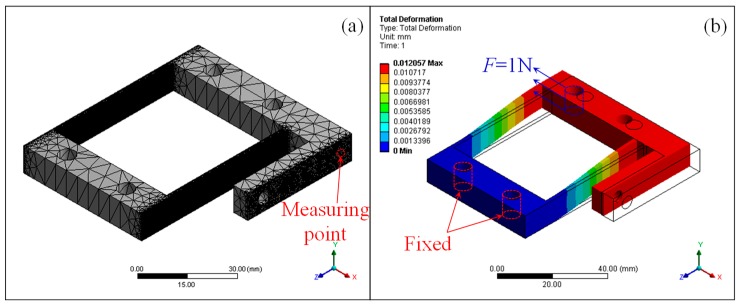
Finite element analysis of the flexure parallel mechanism using the designed model: (**a**) mesh model; (**b**) boundary conditions and results of deformation.

To evaluate the difference of the stiffness characteristics between the designed model and the as-built LBM flexure parallel mechanism, finite element analysis was utilized to provide verification and comparison. Firstly, finite element analysis was established utilizing the designed 3D model of the flexure parallel mechanism. The material properties of the 316L material provided by the feedstock supplier were applied, i.e., Young’s modulus of 166.2 GPa and Poissons’ ratio of 0.33 (acquired according to GB/T 228.1-2010). The finite element analysis was accomplished through the ANSYS workbench. As shown in Figure 5a, a mesh model comprising more than 437,038 nodes and 255,429 elements was set up. Boundary conditions that were identical to the experimental setup were applied and the results of deformation were obtained as shown in Figure 5b. The stiffness of the designed flexure parallel mechanism was *K*_ideal_ = 83.022 N/mm. The deviation of stiffness of the flexure parallel mechanism between the designed model and the experimental result was around 18.9%. It can be concluded that the ideal geometrical parameters are not suitable for predicting the stiffness factor of the LBM flexure mechanism due to the manufacturing errors. Therefore, a second finite element analysis was performed in which the flexure parallel mechanism with the 3D-scanned dimensional parameters was utilized, while the material properties and the boundary conditions were fixed. The stiffness of the 3D-scanned flexure parallel mechanism was *K*_3D-scanned_ = 81.66 N/mm, which represents a deviation of 16.9%. Though the 3D-scanned geometrical parameters are better than the ideal ones, it is still not accurate for predicting the stiffness of the LBM flexure mechanism. This suggests that the measured geometrical parameters or the material properties are not correct.

**Figure 6:**
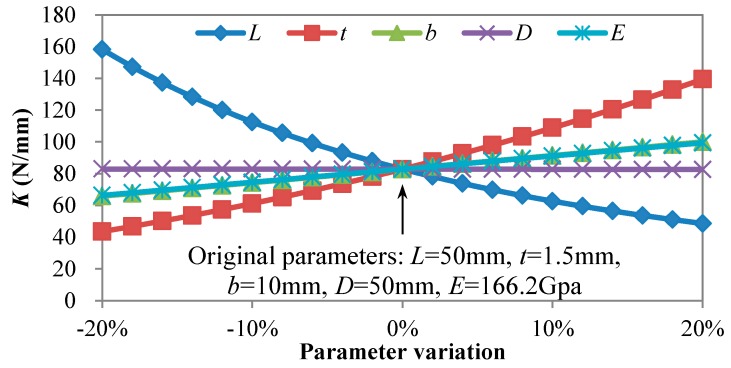
Parameter study of the flexure parallel mechanism through finite element analysis.

Considering the three-dimensional geometrical errors as shown in Section 3.1, a parameters study was performed utilizing finite element analysis to evaluate the key factors for the stiffness of the LBM flexure parallel mechanism. Five factors were taken into consideration, including Young’s modulus *E*, hinge thickness *t*, hinge width *b*, hinge length *L*, and hinge distance *D*. The boundary conditions of the finite element analysis were identical to the former analysis. During the parameters study, variations of each factor from −20% to +20% with reference to the designed value were carried out, while the other factors were kept constant at the designed value. The variation of each factor was divided into 20 increments corresponding to 105 test points. As shown in Figure 6, the resulting variation of stiffness with reference to the five factors was obtained. Within the studied parameter range, it was found that the stiffness decreases as *L* increases or any of *b*, *E*, and *t* decreases, and the hinge distance *D* makes no difference to the stiffness in general. The stiffness is nonlinearly related to the hinge thickness *t* or the hinge length *L*, while it is linearly related to the Young’s modulus *E* or hinge length *L*. It was also discovered that the stiffness is most sensitive to the hinge length *L* among the five factors under the same deviation.

Recalling the worst geometry deviation of ±0.165 mm as detailed in Section 3.1, the worst situations of deviation for the four dimensional parameters were ±0.3%, ±11%, ±1.7%, and ±0.3% for *L*, *t*, *b*, and *D*, respectively. In addition, it was obtained from the feedstock supplier that the deviation of the Young’s modulus is within 3%. It could be concluded that the deviation between the experimental stiffness factor and the designed model was mainly caused by the deviation of the hinge thickness *t*. The effective hinge thickness value should be smaller than the 3D-measured value.

By decreasing the hinge thickness gradually to 1.43 mm, the stiffness decreases to *K*_effective_ = 70.50 N/mm, and the deviation with respect to the experimental results decreases to 1%. Hence, the effective ratio of hinge thickness can be determined to be around 0.95. It can be concluded that the detected hinge thickness through 3D scanning is not accurate. The error mainly comes from the rough surface of the as-built hinge and the low accuracy of the 3D scanning. The differences between these stiffness factors are illustrated in Figure 4.

The stiffness characteristics of the flexure mechanism are one of the most distinguishing properties compared with most of the existing AM structural parts. The stiffness factor can help to evaluate the geometry accuracy of the flexure structure and the performance of the manufacturing system indirectly. As shown in Table 2, the obtained effective ratio of hinge thickness for the metallic additive-manufactured leaf-type flexure hinges was compared between those reported in the literature. From the perspective of the manufacturing performance, it was found that the LBM additive-manufactured 316L leaf-type flexure hinges could have smaller thickness deviation with respect to the designed value than that of the EBM additive-manufactured Ti-6Al-4V ones. Such comparison is limited by the rare reports of flexure mechanisms by AM of metal material.

### 3.3. Analysis of Motion Accuracy

**Figure 7:**
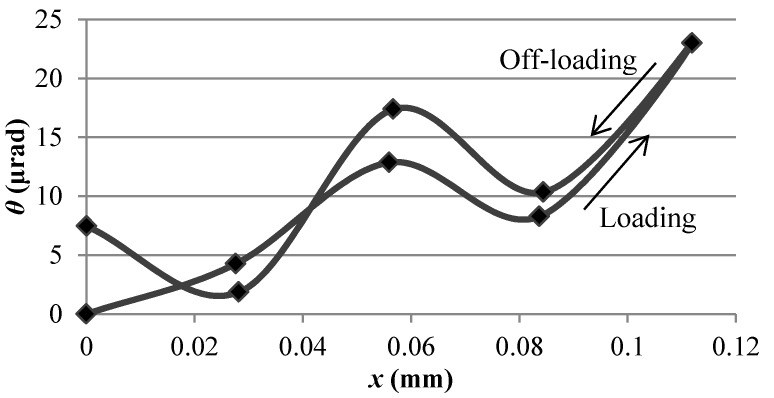
Measured parasitic rotations of the moving block of the LBM additive-manufactured flexure parallel mechanism with reference to the dominating translational displacements.

Generally, the flexure parallel mechanisms could serve as an ultra-precise linear guide endowed with dominating single translational movement. However, parasitic motion, which mainly includes the planar rotation of the moving end, may occur due to manufacturing errors. During the stiffness experiments, the displacements of two points on the moving block of the flexure parallel mechanism were recorded in order to obtain the planar rotations of the moving block. The planar rotations of the moving block, corresponding to the parasitic motions of the LBM additive-manufactured flexure parallel mechanism, against the dominating translational displacement *x* are shown in Figure 7. It was found that the parasitic motions rose up gradually with fluctuation as the dominating translational motions increased. The maximum rotation of the moving block was within 23 μrad, corresponding to a maximum deviation of 0.67 μm between the two sensors under the maximum translational displacement of 111.8 μm. It could be concluded that the presented LBM additive-manufactured flexure parallel mechanism has little parasitic motion. This further verifies the feasibility and reliability of the LBM process for developing flexure mechanisms for precision positioning.

## 4. Discussion

It can be concluded from the results that the presented LBM additive-manufactured flexure parallel mechanism has the linear stiffness characteristics of flexure mechanisms fabricated by traditional processes such as wire-EDM. However, additional issues related to manufacturing errors, material properties, and process parameters should be taken into consideration when developing flexure mechanisms intended for metallic additive manufacturing. For instance, the Young’s modulus is currently not a compulsory parameter in the material data sheet for AM processing. However, AM flexure mechanisms are influenced by both the strength and stiffness (Young’s modulus) of materials. Due to the layer-by-layer formation of the AM process, the as-built flexure hinges may have different stiffness characteristics compared with the designed ones. Process experiments are necessary for developing additive-manufactured flexure mechanisms using metallic materials. A process database is helpful for widespread application.

Due to the high temperature gradient, the thermal stress during the process and the residual stress after the process are two of the main issues for the LBM process. The geometrical errors of the presented LBM flexure sample are mainly caused by the thermal stress during the process since the annealing could relax most of the residual stress after the process. The overall dimensional error of the presented LBM flexure sample was investigated through 3D scanning. It was found that 77% of the geometry deviations were within ±0.093 mm and 93% of the geometry deviations were within ±0.165 mm. For the most sensitive parameters, the dimensional error of hinge thickness *t* was determined as 5% through stiffness experiments. The effective ratio of hinge thickness of the presented LBM flexure hinge is much better than that in existing research on EBM flexures. However, the dimension accuracy of the structure before and after cutting off from the plate has not been compared. The relationship between the thermal stress and the building error of the flexures suggests a direction for future research.

The manufacturing errors of the additive-manufactured flexure mechanisms are distributed in three-dimensional space. The freeform fabrication of parts introduces three-dimensional manufacturing errors even to the planar additive-manufactured flexure hinges as presented. Though the dimensional error of the presented LBM flexure mechanism did not ruin its applicability as designed, the influences of the process parameters to the fabrication quality and material properties of the flexure hinges have not been covered in this article but will be in future works. Some of the important process parameters may include building direction, laser power, scanning speed, etc. In order to achieve ultra-precise positioning, error analysis and closed-loop control are necessary to manipulate systems based on metallic additive-manufactured flexure mechanisms.

The LBM technique can be applied to flexure mechanisms with complex geometries. However, extra care is necessary to make LBM flexure parts more feasible for engineering applications. Generally, issues regarding material properties, geometric constraints, and postprocessing when changing from traditional subtractive manufacturing to additive manufacturing should be considered as well. Typically, the manufacturing accuracy and the surface quality make significant differences to the static characteristics and the dynamic characteristics of the flexure mechanisms, respectively. In order to reduce or eliminate the geometric deformation caused by thermal residual stress during the LBM process, further studies on modeling, simulation, and improvement would be helpful. In addition, since the flexure mechanisms work based on the deformation of the flexure parts, fatigue life is critical to the surface quality. Hence, future works should also focus on investigating and improving the surface quality of the LBM flexure parts.

## 5. Conclusions

In this study, a flexure parallel mechanism was fabricated, tested, and analyzed base on LBM additive manufacturing using 316L stainless-steel material. Three-dimensional manufacturing errors of the as-built flexure part were observed and characterized using 3D scanning. It was found that the presented LBM additive-manufactured flexure parallel mechanism had linear stiffness during the experiments, but deviations were observed as compared with the designed model. Consequently, a parametric study was utilized to determine the key factor for the stiffness deviation. Then, the effective thickness of the LBM leaf-type flexure hinge was determined to be around 0.95, which is better than that of EBM flexure hinges in existing studies. Finally, the parasitic motions of the LBM additive-manufactured flexure parallel mechanism were investigated and little parasitic motions was observed. The results suggest that the presented LBM additive-manufactured flexure parallel mechanism is suitable for precision engineering applications and highlights the promising outlook for the LBM of flexure mechanisms. Future works will include the dynamics analysis and fatigue characteristics of metallic additive-manufactured flexure mechanisms for nanopositioning.

## Figures and Tables

**Table 1:** Powder composition of the 316L for the LBM process (wt.%).

Composition	Cr	Ni	Mn	Si	O	Cu	P	C	Fe
Weight	17.6	12.4	1.26	0.49	0.056	0.19	0.01	0.018	Balance

**Table 2:** Comparison of effective ratio of hinge thickness for metallic additive-manufactured leaf-type flexure hinges.

AM Process and Material	EBM, Ti-6Al-4V			LBM, 316L
Research Group	Merriam and Howell [14,16]	Pham [17]	Fiaz [18]	This Article
Effective Ratio	0.83	1.27	0.73–0.76	0.95
